# Investigation of the glucocorticoid receptor co-chaperone FKBP5 in individuals with first-episode psychosis

**DOI:** 10.1192/j.eurpsy.2021.1907

**Published:** 2021-08-13

**Authors:** D. Theodoridou, A. M. Vlaikou, A. Karampas, G. Georgiou, M.D. Filiou, M. Syrrou, P. Petrikis

**Affiliations:** 1 Laboratory of Biology, Faculty of Medicine, School of Health Sciences, University of Ioannina, Ioannina, Greece; 2 Laboratory of Biochemistry, Department of Biological Applications and Technology, School of Health Sciences, University of Ioannina, Ioannina, Greece; 3 Biomedical Research Division, Institute of Molecular Biology and Biotechnology, Foundation of Research and Technology Hellas (IMBB-FORTH), Ioannina, Greece; 4 Department of Psychiatry, Faculty of Medicine, School of Health Sciences, University of Ioannina, Ioannina, Greece

**Keywords:** glucocorticoids, FKBP5, stress, First episode psychosis

## Abstract

**Introduction:**

Stress has been associated with the onset and progression of neuropsychiatric conditions. The neuroendocrine response to psychosocial stressors is mediated via the hypothalamus-pituitary-adrenal axis, resulting in systemic glucocorticoid secretion. FKBP5 is a co-chaperone of the cortisol-bound glucocorticoid receptor. FKBP5 Single Nucleotide Polymorphisms (SNPs) may indicate stress-response alterations, thus affecting vulnerability or resilience to neuropsychiatric phenotypes.

**Objectives:**

To investigate the FKBP5 polymorphism rs1360780 and FKBP5 mRNA levels in a well-characterized, drug-naïve sample of First-Episode Psychosis (FEP) individuals and matched controls.

**Methods:**

For genotyping rs1360780, whole blood DNA was extracted from FEP individuals and matched controls. The presence of the C (protective)→T (risk) alleles was assessed using TaqMan SNP genotyping assay. Peripheral Blood Mononuclear Cells (PBMCs) were isolated and whole RNA was extracted. FKBP5 mRNA levels were detected with RT-qPCR, using SYBRgreen. Results were normalized against the 18s rRNA reference gene. Statistical analysis was performed in GraphPad Prism 8.

**Results:**

The distribution of C→T alleles of rs1360780 genotyped in FEP (N=44) and controls (N=39) indicate a statistically significant prevalence of the C/C alleles in FEP individuals (*p=0.0432). mRNA FKBP5 data revealed increased levels of FKBP5 in FEP individuals (N=25) compared to controls (N=18), (***p=0.0007).

**Conclusions:**

Our data show increased FKBP5 mRNA levels in FEP individuals compared to matched controls, as well as the presence of the rs1360780 protective (C) allele. Follow up studies include investigation of the translational profile of stress-mediators, in order to pave an individualized approach towards deciphering psychosis onset pathobiology.

**Disclosure:**

No significant relationships.

